# Cusp Catastrophe Polynomial Model: Power and Sample Size Estimation

**DOI:** 10.4236/ojs.2014.410076

**Published:** 2014-11-18

**Authors:** Ding-Geng(Din) Chen, Xinguang(Jim) Chen, Feng Lin, Wan Tang, Y. L. Lio, (Tammy) Yuanyuan Guo

**Affiliations:** 1 Center of Research, School of Nursing, University of Rochester Medical Center, Rochester, USA; 2 Department of Biostatistics and Computational Biology, University of Rochester Medical Center, Rochester, USA; 3 Institute of Data Sciences, University of Rochester, Rochester, USA; 4 Department of Epidemiology, University of Florida, Gainesville, FL, USA; 5 Wuhan University School of Public Health, Wuhan, China; 6 AD-CARE, Department of Psychiatry, University of Rochester Medical Center, Rochester, NY, USA; 7 Department of Mathematical Sciences, University of South Dakota, Vermillion, SD, USA; 8 Department of Statistics, Central South University, Changsha, Hunan, P. R. China

**Keywords:** Cusp catastrophe model, Polynomial regression method, Statistical power analysis, Sample size determination

## Abstract

Guastello’s polynomial regression method for solving cusp catastrophe model has been widely applied to analyze nonlinear behavior outcomes. However, no statistical power analysis for this modeling approach has been reported probably due to the complex nature of the cusp catastrophe model. Since statistical power analysis is essential for research design, we propose a novel method in this paper to fill in the gap. The method is simulation-based and can be used to calculate statistical power and sample size when Guastello’s polynomial regression method is used to cusp catastrophe modeling analysis. With this novel approach, a power curve is produced first to depict the relationship between statistical power and samples size under different model specifications. This power curve is then used to determine sample size required for specified statistical power. We verify the method first through four scenarios generated through Monte Carlo simulations, and followed by an application of the method with real published data in modeling early sexual initiation among young adolescents. Findings of our study suggest that this simulation-based power analysis method can be used to estimate sample size and statistical power for Guastello’s polynomial regression method in cusp catastrophe modeling.

## 1. Introduction

Popularized in the 1970’s by Thom [1], Thom and Fowler [2], Cobb and Ragade [3], Cobb and Watson [4], and Cobb and Zack [5], catastrophe theory was proposed to understand a complicated set of behaviors including both gradual and continuous changes and sudden and discrete or catastrophical changes. Computationally, there are two directions to implement this theoretical catastrophe theory. One direction is operationalized by Guastello [6,7] with the implementation into a polynomial regression approach and another direction by a stochastic cusp catastrophe model from Cobb and his colleagues [5] with implementation in an R package in [8]. And this paper is to discuss the first direction on polynomial cusp catastrophe regression model due to its relative simplicity and easy for implementation as simple regression approach. This model has been used extensively in research. Typical examples include modeling of accident process [7], adolescent alcohol use [9], changes in adolescent substance use [10], binge drinking among college students [11], sexual initiation among young adolescents [12], nursing turnover [13], and effect of HIV prevention among adolescents [12,14].

Even though this polynomial regression method has been widely applied in behavioral studies to investigate the existence of cusp catastrophe, to the best of our knowledge, no reported research has addressed the determination of sample size and statistical power for this analytical approach. Statistical power analysis is an essential part for researchers to efficiently plan and design a research project as pointed out in [15,16,17]. To assist and enhance application of the polynomial regression method in behavioral research, this paper is aimed to fill this method gap by reporting the Monte-Carlo simulation-based method we developed to conduct power analysis and to determine sample size.

The structure of the paper is as follows. We start with a brief review of the cusp catastrophe model (Section 2), followed by reporting our development of the novel simulation-based approach to calculate the statistical power (Section 3). This approach is then verified through Monte Carlo simulations and is further illustrated with data derived from published study (Section 4). Conclusions and discussions are given at the end of the paper (Section 5).

## 2. Cusp Catastrophe Model

### 2.1. Overview

**Figure 1:**
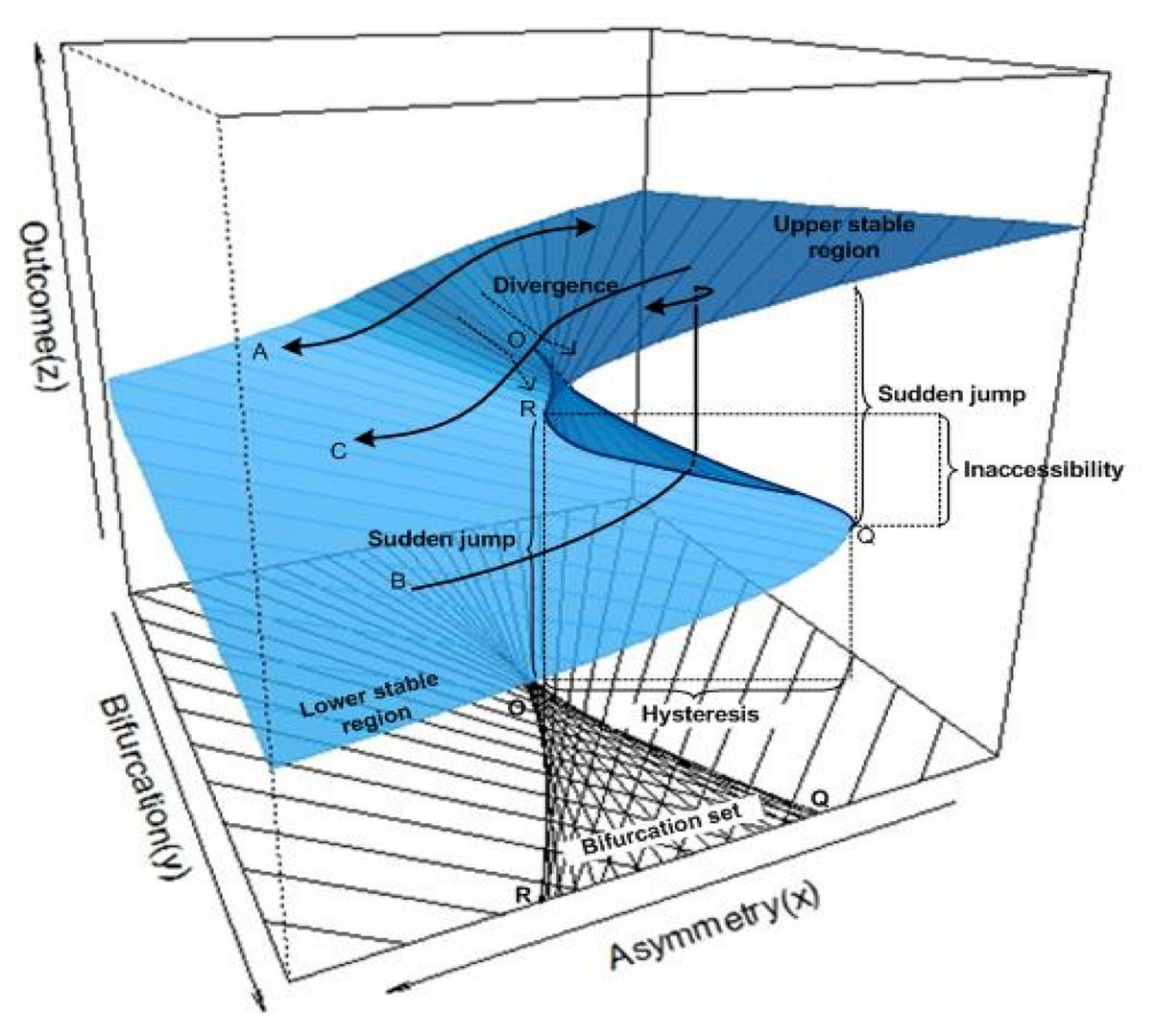
Cusp catastrophe model for outcome measures (*Z*) in the equilibrium plane with asymmetry control variable (*X*) and bifurcation control variable (*Y*). (Annotated by the authors with the original graph produced by Grasman’s R package “cusp”)

The cusp catastrophe model is proposed to model system outcomes which can incorporate the linear model with extension to nonlinear model along with discontinuous transitions in equilibrium states as control variables vary. According to the catastrophe systems theory [1,18,19,20], the dynamics for a cusp system outcome is expressed by the time derivative of its state variable (often called behavioral variable within the context of catastrophe theory) to the potential function: *V*(*z;x,y*) = 1/4 *z*^4^ −1/2 *z*^2^*y* − *zx*. The first derivative of V will consist of the equilibrium plane of the cusp catastrophe:

(1) $$\partial V(z,x,y)/\partial z=z^{3}-yz-x=0,$$

where *x* is called asymmetry or normal control variable and *y* is called bifurcation or splitting control variable. In the model, the two control variables *x* and *y* co-vary to determine the behavior outcome variable *z*. Figure 1 depicts the equilibrium plane which reflects the response surface of the outcome measure (*z*) at various combinations of *x* and *y*. It can be seen from the figure that the dynamic changes in a behavior measure (*z*) has two stable regions (attractors), the lower area in the front left and the upper areas in the front right. Beyond these two regions, behavior *z* becomes unstable. This characteristic can be further revealed by projecting the unstable region to the *x* and *y* control plane as a cusp region. The cusp region is characterized by two lines, line O-Q (the ascending threshold) and line O-R (the descending threshold) of the equilibrium surface. In this region, the outcome measure becomes highly unstable, and sudden change or jumping in behavior status will occur, because a very small change in *x* or *y* or both will lead z to cross either the threshold line O-Q or O-R.

Furthermore, the paths A, B, and C in Figure 1 depict three typical but different pathways of change in the outcome measure (*z*). Path A shows that in any situations where y<O, there is a smooth relation between outcome measure (*z*) and the asymmetry variable (*x*); path B shows that in any situations where *y*>O, if the asymmetry variable *x* increases to reach and pass the ascending threshold link O-Q, outcome measure (z) will increase suddenly from the low stable region to the upper stable region of the equilibrium plane; and Path C shows a sudden drop in outcome measure (*z*) as *x* declines to reach and pass the descending threshold line O-R.

From the affirmative description, it is clearly that a cusp model differs from a linear model in that: (1) A cusp model allows the forward and backward progression follows different paths in the outcome measure and both processes can be modeled simultaneously (see Paths B and C in Figure 1) while a linear model only permits one type of relationship; (2) A cusp model covers both a discrete component and a continuous component of a behavior change while a linear model covers on continuous process (Path A). In this case a linear model can be considered as a special case of the cusp model; (3) A cusp model consists of two stable regions and two thresholds for sudden and discrete changes. Therefore, the application of the cusp modeling will advance the linear approach and better assist researchers to describe the behavior data while evidence obtained from such analysis, in turn, can be used to advance theories and models to better explain a behavior.

### 2.2. Guastello’s Cusp Catastrophe Polynomial Regression Model

To operationalize the cusp catastrophe model for behavior research, Guastello [6,7] developed the polynomial regression approach to implement the concept of cusp model. Since the first publication of this method, it has been widely used in analyzing real data as we described in the Introduction. In this study, we referred the method as Gastello’s polynomial cusp regression. According to Gustello, this approach is derived by inserting regression β coefficients into the Equation (1), with change scores Δz = z_2_ − z_1_ (the differences in the measurement scores of a behavior assessed at time 1 and time 2) as a numerical approximation of dz:

(2) $$\Delta z=\beta_{0}+\beta_{1}\times {z_{1}}^{3}+\beta_{2}\times {z_{1}}^{2}+\beta_{3}\times y\times z_{1}+\beta_{4}\times x+\;\;\;+\beta_{5}\times y+\varepsilon ,$$

where β_0_ is the intercept and ε is the normally distributed error term. Two additional term β_2_ × z_1_^2^ and β_5_ × y are added to capture potential deviations of the data from the equilibrium plane. When conducting modeling analysis, a cusp is indicated ONLY if the estimated β_1_ for the cubic term, plus β_3_ (for the interaction term) or β_4_ (for control variable *x*) in Equation 2 are statistically significant.

To demonstrate the efficiency of the polynomial regression approach in describing behavioral changes that are cusp, Guastelly [7] recommended a comparative approach. In this approach, two types, four alternative linear models can be constructed and used in modeling the same variables:

1. Change scores linear models
  (3) $$\Delta z=\beta_{0}+\beta_{1}z_{1}+\beta_{4}\;x+\beta_{5}\;y$$
  (4) $$\Delta z=\beta_{0}+\beta_{1}z_{1}+\beta_{3}\;{\text{yz}}_{1}+\beta_{4}x+\beta_{5}\;y$$
2. Pre-and post- linear models
  (5) $$z_{2}=\beta_{0}+\beta_{1}z_{1}+\beta_{4}\;x+\beta_{5}\;y$$
  (6) $$z_{2}=\beta_{0}+\beta_{1}z_{1}+\beta_{3}\;{\text{yz}}_{1}+\beta_{4}\;x+\;\;\;+\beta_{5}\;y$$

These alternative linear models add another analytical strategy to strength the polynomial regression method. A better data-model fitting (or a larger R^2^) of the cusp model (2) than the alternative linear models (3) through (6) is often used as additional evidence supporting the hypothesis that the dynamics of a study behavior follows the cusp catastrophe. Fitting Guastello’s cusp regression model and the four alternative models can all be conducted with commonly available statistical software, including SAS, SPSS, STATA and R. More recent discussions and applications of the cusp catastrophe modeling methods can be found in [21].

## 3. Simulation-Based Power Analysis Approach for Guastello’s Cusp Regression

### 3.1. A Brief Introduction to Statistical Power

In statistics, power is defined as the probability of correctly rejecting the null hypothesis. Stated in common language, power is the fraction of the times that the specified null-hypothesis value will be rejected from statistical tests. Operationally based on this definition, if we specify an alternative hypothesis H_1_, a desired type-I error rate α, and a desired power (1 − β), then we can calculate the required sample size n. Alternatively, we can calculate the statistical power (1 − β) as a function of sample size n under a specified alternative hypothesis H_1_ and a desired type-I error rate α. There are extensive literatures on sample size calculation as well as statistical power analysis, see the seminal books from [15,16,17] for power analysis for behavioral sciences.

As detailed in Chapter 7 in [17], five factors related to research design interplay with each other to determine the statistical power and sample size for a simple t-test: 1) the rate of type-I error α, 2) the desired statistical power 1 − β, 3) the expected treatment effect size of δ, 4) the standard error s^2^ for the expected effect size, and 5) the sample size n. The mathematical formula can then be derived as n ≥ 2(s^2^/δ^2^)[z_1−α_+z_1−β_]^2^. Therefore, to determine the required sample size n, we would need to provide data for four of the five design characteristics. Typically, the type-I error α is set at 0.05 and the desired power (1 − β) is chosen to be 0.85 (or 0.80). The other two will be treatment effect size δ and its standard error s^2^. Depending on actual research questions, different values are often selected for these two characteristics.

Extending the same concept described above for Guastello’s polynomial cusp regression, we would need to specify the corresponding parameter effect size for all βs in Equation (2), the standard deviation of the error term ε. In addition, we need to specify the distribution of the two control variables, the asymmetry *x* and the bifurcation *y*; and the distribution of the outcome variable z at time 1 (i.e. *z*_1_). With these parameters and variables being specified, the required sample size for a significant Cuastello’s cusp regression model can be determined and statistical power can be analyzed.

### 3.2. Simulation-Based Approach for Power Analysis and Sample Size Determination

Power analysis and sample size determination can be developed for specific purpose. Typically, it is developed to detect treatment effect as in clinical trials or to detect the effect of specific risk factor as in regression. Similar development can be done to Guastello’s cusp regression model for specific repressor in asymmetry variable (*x*) or the bifurcation variable (*y*) if they are linked to multiple regressors or even to the overall goodness-of-fit index of R^2^. However, we aim to tackle a more complicated problem to determine whether we can detect a significant overall cusp model. The complexity of cusp catastrophe model makes it rather challenging, if not impossible to derive an analytical formula to determine the statistical power for Guastello’s cusp regression. To deal with this difficult, we propose a Monte-Carlo simulation-based approach. In this method the statistical power is calculated as the fraction of the times that the specified null-hypothesis of “no cusp” is rejected at the given level of type I error. Stated in another way, if there is a cusp, the statistical power will be, among 100 simulations, how many times can we detect the cusp given the sample size and type I error? The detailed steps of the simulation-based approach are outlined as follows:

1. Simulate data with sample size (n) (i.e. the number of observations for Guastello’s cusp regression modeling) for the asymmetry variable *x*, bifurcation variable *y* and outcome variable at time 1 (i.e. z_1_). Data are generated under required specifications for desired study, such as normal distribution with specific means and standard deviations. Guastello’s cusp regression requires that all variables be standardized before data analysis and modeling. In this case, the standard normal distribution can be used to generate data for *x*, *y* and *z*_1_;
2. Specify model parameter effect size β = (β_0_, β_1_, β_2_, β_3_, β_4_, β_5_) and the standard deviation σ of the error term of ε (Equation 2) obtained from prior knowledge;
3. Calculate z_2_ = z_1_ + β_0_ + β_1_z_1_^3^ + β_2_z_1_^2^ + β_3_ y z_1_ + β_4_ x + β_5_ y + ε using the data obtained in the previous two Steps. Also generate Δz= z_2_ − z_1_;
4. Fit the Guastello’s cusp regression model (Equation 2) with least squares method using the data generated for Δz, x, y, and z_1_. After model fitting, a significant test is conducted to determine whether the data fit Guastello’s cusp regression model satisfactorily according to the decision rules proposed by Guastello (1982): (1) the estimated β1 for the cubic term and (2) β_3_ (for the y and z_1_ interaction term) or β_4_ (for control variable x) must be are statistically significant;
5. Repeat Steps 1 to 4 a large number of times (typically 1,000) and calculate the proportion of simulations which satisfy the Guastello’s decision rules. This proportion then provides an estimate of the statistical power for the pre-specified sample size and the study specifications given in Steps 1 and 2;
6. With the above established five steps for power assessment, sample size is then determined to reach a pre-specified level of statistical power. This is carried out by running Steps 1 to 5 with a range of sample sizes (n) first to obtain the corresponding values of statistical power. Then a statistical power curve is constructed for these ranges of sample sizes. With this power curve, the sample size is determined through back-calculation for a pre-specified power, such as power = 0.85.

The simulation-based approach described above is implemented in free R package and the computer program is available up request from the authors.

## 4. Simulation Study and Real Example

### 4.1. Monte-Carlo Simulation Analysis

#### 4.1.1. Rationale

To verify the novel approach proposed in Section 3, we simulated four scenarios with n = 100 observations for each using Guastello’s cusp polynomial regression model (2). The four scenarios represent four cases of σ with different measurement errors (i.e. σ =1, 2, 3, and 4). We hypothesized that data with smaller measurement errors will fit the cusp model better than the data with larger errors if the Guastello’s cusp polynomial regression method is used to detect cusp catastrophic changes. Consequently, a larger sample size would be needed to detect a cusp for data with greater measurement errors.

#### 4.1.2. Data Generation

Data are generated with the asymmetry variable x, bifurcation variable y and outcome variable at time 1 (i.e. z_1_) being set as standard normal distribution. The parameter effect size vector is set as β = (β_0_, β_1_, β_2_, β_3_, β_4_, β_5_) = (0.5, 0.5, 0.5, 0.5, 0.5, 0.5). To illustrate the impact of measurement errors on sample sizes, we generate the error term ε following the normal distribution as ε ~ N(0, σ^2^) with increasing measurement error standard deviation of σ = 1, 2, 3, and 4 for each of the four scenarios.

With the generated x, y and z1 along with the input values of β and σ, Δz is generated using the Guastello’s polynomial regression model. This is achieved by plugging in all values of x, y, z_1_, β, σ and ε into the following equation:

$$\Delta z=\beta_{0}+\beta_{1}{z_{1}}^{3}+\beta_{2}{z_{1}}^{2}+\beta_{3}\;y\;z_{1}+\beta_{4}\;x+\;\;\;+\beta_{5}\;y+\varepsilon .$$

**Figure 2:**
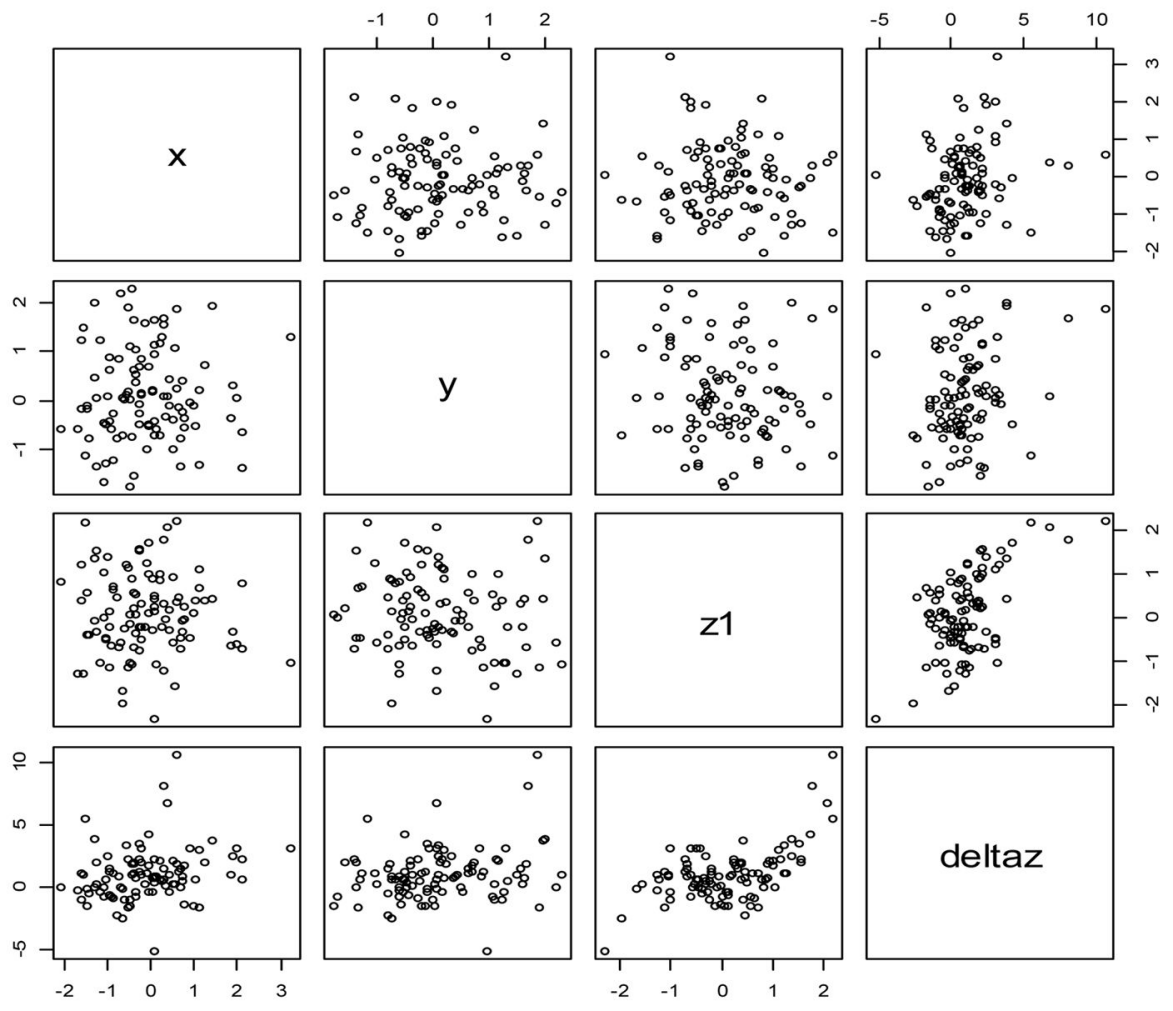
Example of simulated data when σ =1 where the distributions of *x*, *y*, *z*_1_ are standard normal (the upper left 3 by 3 plots) and the relationships between Δz to *x* (as linear), to *y* (as linear) and to *z*_1_ (as cubic).

Figure 2 illustrates one realization of the data generation with σ = 1 in a pair plot. It can be seen from the figure that the distributions for x, y and z^1^ are random (the upper left 3 by 3 plots). Furthermore, Δz is linearly related to x as seen from the upper right plot. The second plot on the right-side illustrates the linear relationship between Δz and y under fixed z^1^ and the third plot on the right-side illustrates the cubic relationship between Δz and z1. For σ = 2, 3, and 4 (data not shown in figure), the corresponding pair plots would have larger variations.

#### 4.1.3. simulation Analysis

Four data sets for the four scenarios (e.g., σ = 1, 2, 3, and 4) are simulated first. The simulated data are then fitted with Guastello’s cusp regression model using least squares method. The summary statistics of the analyses are given in Table 1. It can be seen from the table that for the Scenario where σ = 1, all the parameters of the polynomial regression model are statistically highly significant (p < 0.001) with R^2^ = 0.763, indicating adequate data-cusp model fitting and F-statistic = 60.71 indicating highly significance of the polynomial regression model. The estimated σ̂ = 1.053, slightly greater than the true σ = 1. Since β_1_, β_3_ and β_4_ are all highly significant, we conclude that the Guastello’s polynomial regression method is sufficient to detect the specified cusp.

Results of other three scenarios in Table 1 indicate that as σ increases, the goodness of data-model fitting declines. In the scenario where σ = 2, the R^2^ drops to 0.454, F-statistic drops to 15.61 (still significant), and the estimated σ = 2.107, close to the true σ = 2. In this case, both β_1_ and β_3_ remain significant, indicating the existence of a cusp. With regard to Scenario 3 where σ = 3, the R^2^ further drops to 0.278 and F-statistic to 7.227. The estimated σ = 3.160, again close to its true σ = 3. In this case, only β_1_ is highly significant and β_3_ marginally significant, indicating that a cusp is likely. In Scenario 4 where σ = 4, none of the estimated parameters required to support the cusp is statistically significant. Therefore, we could not be able to determine if the data contain a cusp. A power analysis is needed to assess if the sample size (n = 100) is adequate.

#### 4.1.4. Sample Size Estimation

**Figure 3:**
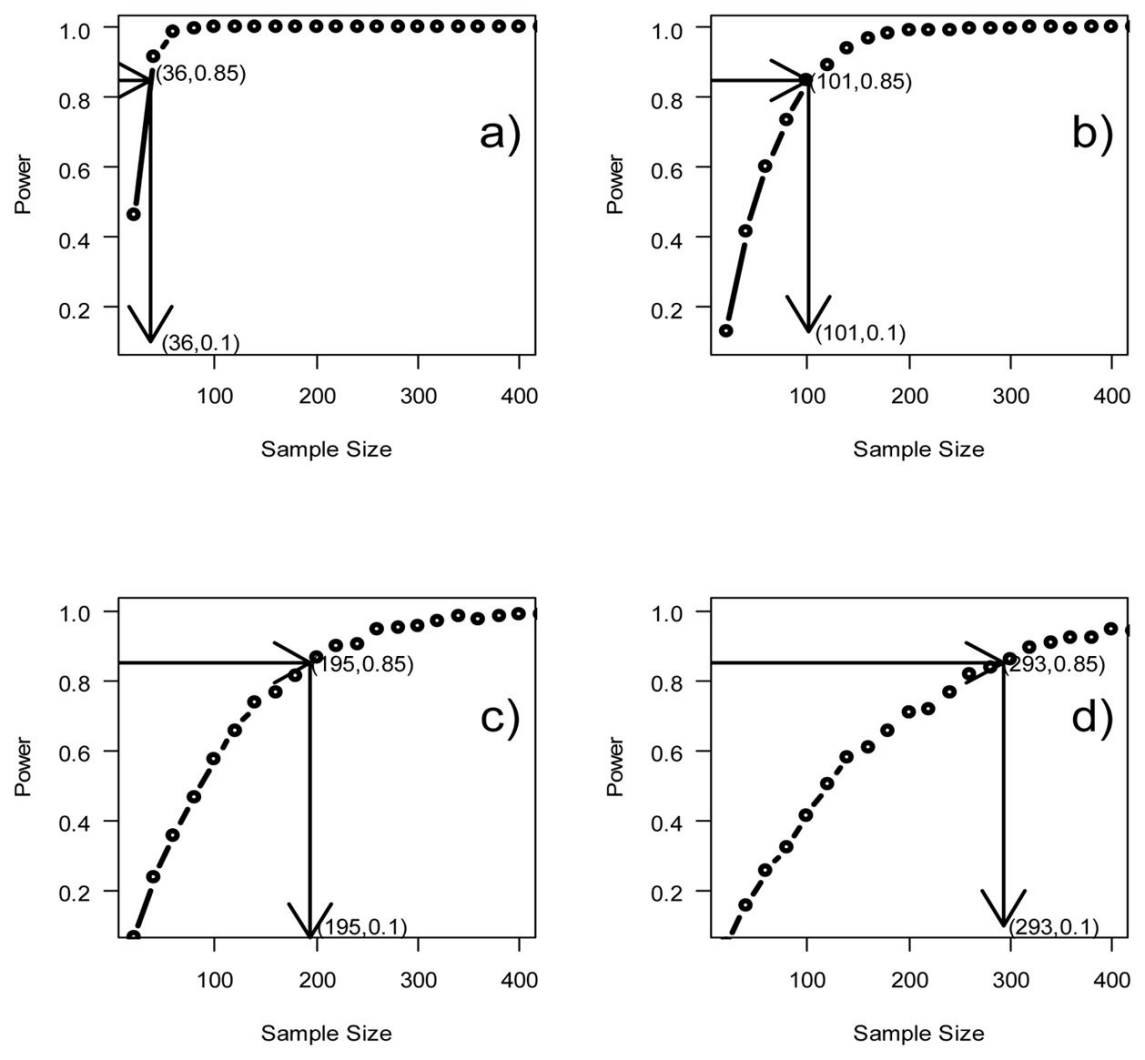
Statistical power curves corresponding to σ = 1 in plot a), σ = 2 in plot b), σ = 3 in plot c) and σ = 4 in plot d). The arrows illustrate the sample size determination from power of 0.85 to calculate the sample size required.

To demonstrate the proposed novel simulation method, we estimate sample sizes needed for each of the four scenarios to achieve 85% statistical power employing this method and the estimated parameter β = (β_0_, β_1_, β_2_, β_3_, β_4_, β_5_) and the estimated σ from Table 1 in the previous step. Figure 3 summarizes the results. Data in Figure 3 indicate that with 85% statistical power to detect the underlying cusp, the required sample sizes for Scenarios 1 through 4 are 36, 101, 195 and 293, respectively. The required sample size varies proportionately with measurement errors. This result adds more evidence supporting the validity of the simulation-based approach we proposed for power analysis.

#### 4.1.5. Reverse-Verification

If the novel simulation-based approach is valid, the sample size estimates for each of the four scenarios described in previous section will allow approximately 85% chance to detect the underlying cusp. Therefore, we took a reverse approach to compute statistical power by applying the calculated sample size as input for each of the four scenarios. Results in Figure 3 indicated that for Scenario 1, a sample size of 36 observations will be adequate to detect the cusp with 85% statistical power.

To demonstrate this result, we make use Monte-Carlo procedure and randomly sample 36 observations from the simulate data (n = 100) used for Scenario 1 (σ = 1). We then fit the data to the Guastello’s cusp regression model. We use the same criteria (significant β_1_, plus either β_3_ or β_4_) to assess the detection of a cusp. Among 1000 repeats of the Monte-Carlo simulations with sample size n=36, we found 833 times (83.3%) significant. This result indicates that the power analysis of the simulation method we proposed is close to 85%. In another word, the method we proposed is slightly conservative, which is good for research design. The template is designed so that author affiliations are not repeated each time for multiple authors of the same affiliation. Please keep your affiliations as succinct as possible (for example, do NOT post your job titles, positions, academic degrees, zip codes, names of building/street/district/province/state, etc.). This template was designed for two affiliations.

### 4.2. Verification with Published Data

The best approach to demonstrate the validity of the simulation approach would be to test it with observed data. To use our approach, we need two sets of data from any reported study: parameter estimates as effect size β = (β_0_, β_1_, β_2_, β_3_, β_4_, β_5_) and estimated mean error of model fitting σ̂. However, we experienced difficulties in finding such data from all the studies we accessed in the published literature database. For example, all β coefficients were reported by all studies but β_0_ was not; furthermore, data-model fitting error fitting σ̂ was never reported in any of the published studies using Guastelle’s cusp polynomial regression method. Fortunately, one author of this paper [12] published a study that modeled early sexual initiation among young adolescents using this polynomial regression approach.

Briefly, in Chen’s study participants were 469 virgins in the control group for a randomized controlled trial to assess the effect of an HIV behavioral prevention intervention program [22,23]. The participants in grade 6 in the Bahamian public schools were randomly assigned to receive either intervention or control conditions. They were followed every 6 months up to 24 months at the time when the analysis was conducted. A participant was categorized as having initiated sex if he or she had the first penile-vagina sexual intercourse during the follow-up period. In addition to sexual initiation, the likelihood to initiate sex was also assessed using a 5-point rating scale with 1 = very unlikely to have sex in the next 6 months and 5 = very likely to have sex. A sexual progression index (SPI) was thus created as the dependent variable for modeling analysis was defined as the first time. SPI = 1 for participants who never had sex and reported very unlikely to have sex; SPI = 2 for participants who never had sex but unsure if they are going to have sex in the next 6 months; SPI = 3 for participants who never had sex but reported very like to have sex in the next 6 months; and SPI = 4 for participants who initiated sex. In addition to SPI, age was used as the asymmetry variable x, and self-efficacy not to have sex (scale score based on 5 items) was used as the bifurcation y.

**Figure 4:**
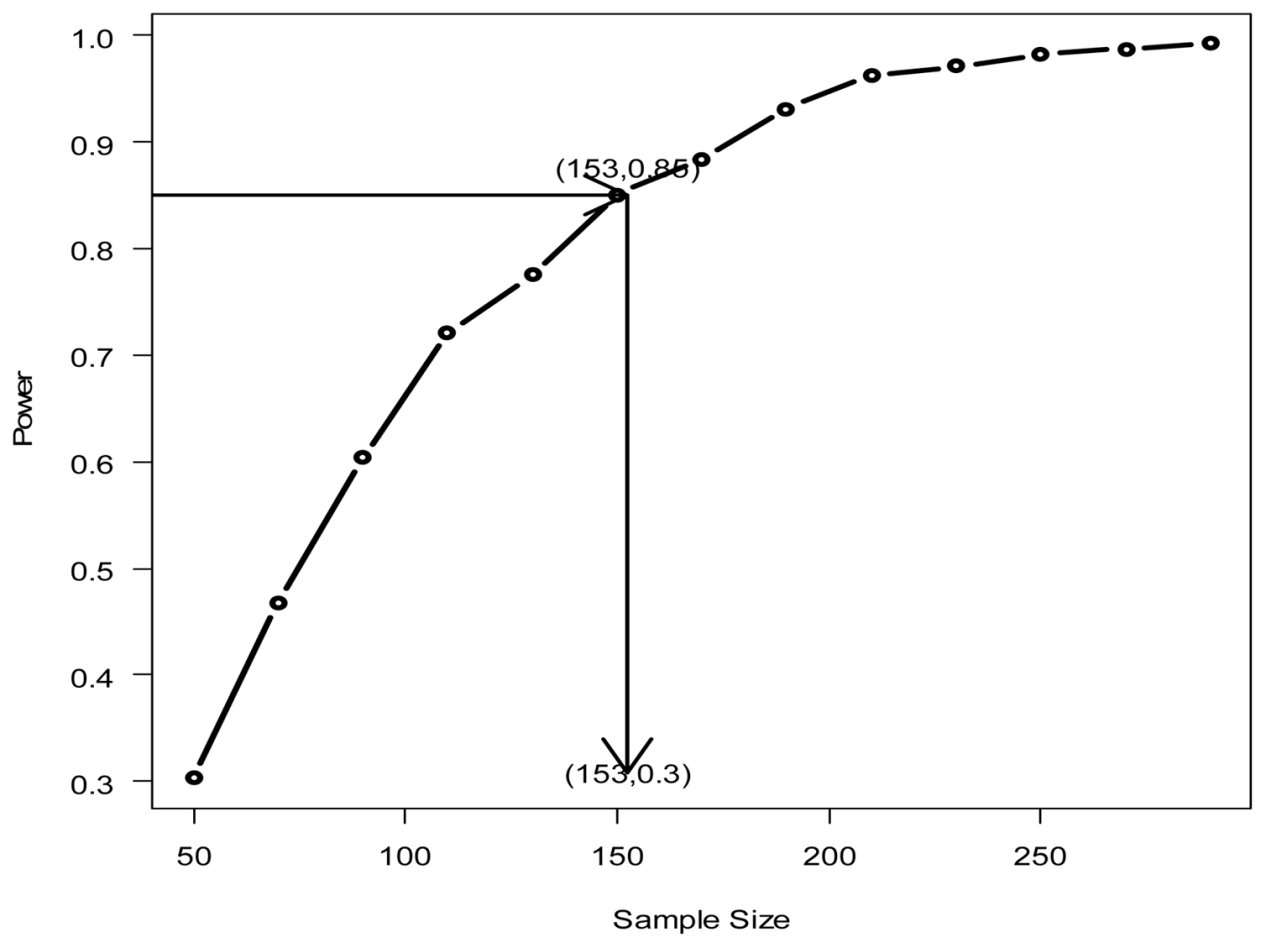
Power curve for Chen et al (2010). The estimated sample size for power of 0.85 is 153

To verify the simulation-based method, the parameter effect size estimates were obtained from the paper with β = (β_0_, β_1_, β_2_, β_3_, β_4_, β_5_)=c(−0.0309,0.0726,−0.4819,−0.1236,0.0613,−0.2693), and the data-model fitting error σ̂ = 0.5033 was obtained by accessing to the original computing records. With these estimates, the simulation-based approach in Section 3.2 is applied. Figure 4 presents the sample size-power curve. From the figure it can be seen that the estimated sample size is 153 to achieve 85% power. This sample size is much smaller than the sample (n=469) in the original study.

## 5. Discussions

In the case where analytical solution to power analysis and sample size determination is difficult, simulation represents an ideal alternative as recommended in [16,17,24]. In this paper, we reported a novel simulation-based approach we developed to estimate the statistical power and to compute sample size for Gustello’s polynomial cusp catastrophe model. The method was developed based on statistical power theory and our understanding of Guastello’s cusp polynomial regression modeling approach. The computing method is programmed using the R software. Results from 1000 repeats of Monte Carlo simulation and empirical data analysis suggest that the method we proposed is valid and can be used in practice to conduct power analysis and to estimate sample size for Guastello polynomial cusp modeling method.

With this approach, researchers can compute statistical power and estimate sample size if they plan to conduct cusp modeling analysis using Gustallo’s polynomial regression method. A detailed introduction to the method can be found in [6,7,21]. Data needed for our methods included parameter effect size estimates for the intercept and five model parameters (β_0_, β_1_, β_2_, β_3_, β_4_, β_5_) and a data-model fitting error σ or its estimate. With the specification of these data, power can be computed for any given sample sizes. In addition to computer power, the commonly used sample size - power curve can be generated to provide a visual presentation between sample size and statistical power. With such power curve, sample size can be estimated for specified power in design and analysis data from cusp catastrophe model.

To make the presentation easier, we confined this novel simulation approach to the situation of one regressor for each control variable in the cusp model. This approach can be easily adopted and extended to multiple regressors for each of the asymmetric (x) and bifurcation (y) variables where the Guastello’s cusp polynomial regression model would need to be extended.

More and more data suggest the utility of cusp modeling approach in characterizing a number of human behaviors, particularly health risk behaviors, such as tobacco smoking, alcohol consumption, hardcore drug use, dating violence, and unprotected sex [10,11,14,21,25,26]. The method we reported in this paper provide an useful tool for researchers to more effectively design their research to investigate these risk behaviors and to assess intervention programs for risk reduction.

By conducting this study, we also noted that previous studies published in the literature do not report adequate information for power analysis. We highly recommend that journal editors ask authors to report all parameter estimates, including β_0_, and data-model fitting error (mean square of error). In addition to power analysis and sample size estimation, such data are also useful for readers to statistically assess appropriateness of the reported results.

There are a number of strengths with the method we presented in this study. The principle and the computing process are not difficult to follow; the data used for the computing can be obtained; the computing software is written with R, available from the authors by request for collaboration; and the computing does not require much time (several seconds to half minutes). We are encouraged on the results from this research and work on extend the results in to stochastic catastrophe model in [4,19]. Despite many advantages, further application of the method in practice is needed.

## Figures and Tables

**Table 1:** Parameter estimates, R^2^, Estimated σ^2^ and F-Statistic from four simulations with σ=1, 2, 3 and 4. The rows bolded are corresponding to the cusp determination.

	σ = 1	σ = 2	σ =3	σ = 4
β_0_ (Intercept)	0.487***	0.473․	0.459	0.446
**β_1_ (z_1_^3^)**	0.540***	0.581***	0.621***	0.661***
β_2_ (z_1_^2^)	0.456***	0.411*	0.367	0.323
β_3_ (y*z_1_)	0.360**	**0.221**	**0.081**	**−0.058**
**β_4_ (x)**	0.563***	0.626**	0.689*	**0.753**
β_5_ (y)	0.468***	0.435․	0.403	0.371
R^2^	0.763	0.454	0.278	0.1856
Estimated σ^2^	1.053	2.107	3.160	4.214
F-Statistic with df = (5, 94)	60.71***	15.61***	7.227***	4.286*

Significant codes:

*** p-value <0.00001,

** p-value <0.001),

* p-value <0.01,

․ p-value<0.05
